# Is Protein Folding a Thermodynamically Unfavorable, Active, Energy-Dependent Process?

**DOI:** 10.3390/ijms23010521

**Published:** 2022-01-04

**Authors:** Irina Sorokina, Arcady R. Mushegian, Eugene V. Koonin

**Affiliations:** 1 Strenic LLC, McLean, VA 22102, USA; 2 Division of Molecular and Cellular Biosciences, National Science Foundation, Alexandria, VA 22314, USA; mushegian2@gmail.com; 3 Clare Hall College, University of Cambridge, Cambridge CB3 9AL, UK; 4 National Center for Biotechnology Information, National Library of Medicine, National Institutes of Health, Bethesda, MD 20894, USA

**Keywords:** protein folding, entropy, free energy, free energy landscape, energy-dependent protein folding, co-translational protein folding, molecular chaperones, physical model of protein folding

## Abstract

The prevailing current view of protein folding is the thermodynamic hypothesis, under which the native folded conformation of a protein corresponds to the global minimum of Gibbs free energy *G*. We question this concept and show that the empirical evidence behind the thermodynamic hypothesis of folding is far from strong. Furthermore, physical theory-based approaches to the prediction of protein folds and their folding pathways so far have invariably failed except for some very small proteins, despite decades of intensive theory development and the enormous increase of computer power. The recent spectacular successes in protein structure prediction owe to evolutionary modeling of amino acid sequence substitutions enhanced by deep learning methods, but even these breakthroughs provide no information on the protein folding mechanisms and pathways. We discuss an alternative view of protein folding, under which the native state of most proteins does not occupy the global free energy minimum, but rather, a local minimum on a fluctuating free energy landscape. We further argue that Δ*G* of folding is likely to be positive for the majority of proteins, which therefore fold into their native conformations only through interactions with the energy-dependent molecular machinery of living cells, in particular, the translation system and chaperones. Accordingly, protein folding should be modeled as it occurs in vivo, that is, as a non-equilibrium, active, energy-dependent process.

## 1. Introduction

For the last six decades, the general understanding in the protein folding field has been that proteins fold into their native conformations driven by decrease in Gibbs free energy (negative Δ*G*). This thermodynamic hypothesis of protein folding stems from the iconic experiments of Anfinsen on in vitro folding of RNase A. Based on the successful refolding of this enzyme into the active, native conformation, Haber and Anfinsen concluded in a seminal 1962 paper that “*the unique secondary and tertiary structure of RNase is, thermodynamically, the most stable configuration*” [1]. Codified in Anfinsen’s 1973 Nobel lecture-based review [2], the thermodynamic hypothesis has become the default physical description of protein folding.

The thermodynamic hypothesis of folding, and in particular, the idea that the native state is the most stable one, that is, the global *G* minimum, is indeed highly attractive and appears natural. Furthermore, this view drastically simplifies theory development and modeling by effectively avoiding the need to explain how and why a protein reaches the unique native conformation: indeed, the global minimum is unique by definition. Assuming that the native conformation occupies a local rather than the global minimum of *G* immediately complicates the problem because this demands an explanation of how this particular minimum is selected among the many other local minima.

In the last two decades, the protein folding problem has been addressed primarily in terms of a free energy landscape that is usually represented as containing a funnel, the bottom of which corresponds to the global *G* minimum, that is, the native conformation; many different shapes of this hypothetical funnel have been considered [3,4,5,6,7,8,9,10,11].

**Figure 1:**
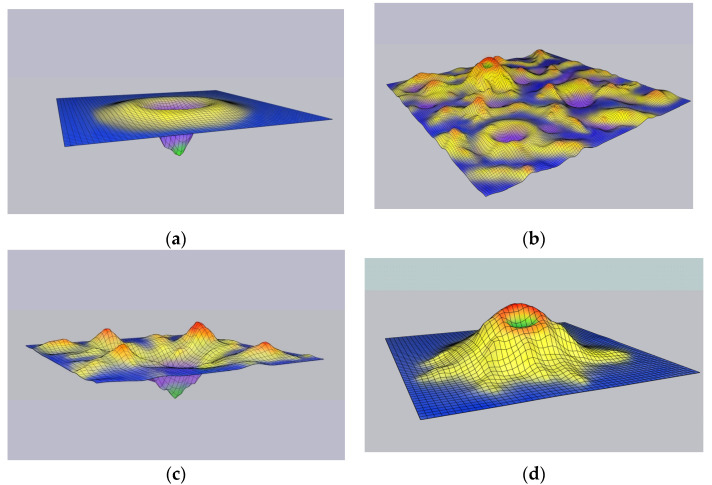
Protein folding energy landscapes in vitro and in vivo. Blue areas are occupied by “perfectly unfolded” conformations with no stable interactions between non-contiguous residues. Yellow and purple areas are populated by more compact protein conformations. Red zones are thought to arise mostly as the result of interactions between the protein and cellular components in a crowded environment. Green zones correspond to proteins in native conformation. (**a**) Canonical funnel-shaped energy landscape that most likely applies only to folding of small, thermodynamically stable proteins as it occurs spontaneously, in vitro, in isolation from all cellular compounds. (**b**) Folding energy landscape for a protein that folds in vivo is poorly understood, but most likely, is complex, rugged, dynamic, and shaped by interactions of the folding polypeptide with multiple cellular components. (**c**) Folding energy landscape of the same small protein as in (**a**) is most likely substantially different and far more complex when folding occurs in a crowded cellular environment. (**d**) Native conformations of most proteins are likely to occupy local thermodynamic minima with higher Gibbs free energy than their unfolded conformations (positive Δ*G* of folding). Such native conformation can only arise as a result of active, energy dependent folding process.

Volcano-shaped landscapes have been also proposed, where during folding, the protein initially has to overcome a barrier of positive Δ*G* due to entropy decrease, but the native structure still occupies the global *G* minimum [12,13] (Figure 1a). However, there is effectively no information on the actual structure of the landscape, and the possibility that the native conformation represents a local minimum in a rugged landscape that is generated and continuously affected by dynamic interactions within the cell environment, rather than the global minimum (Figure 1b), has not been systematically addressed. The distinction between the two classes of models can be formulated, in general terms, as thermodynamic vs. kinetic control of protein folding. Indeed, the early work of Wetlaufer and others (reviewed in [14]) emphasized that the native conformation would be the one with the minimum *G* among the kinetically accessible structures. However, this approach to the study of protein folding has not received much attention or further development, arguably, because it dramatically complicates modeling compared to the straightforward thermodynamic approach.

Although the funnel landscape concept dominates the protein folding field, it is not without its critics. As argued in detail by Ben-Naim, the funnel folding landscape is effectively a metaphor that lacks substantial support [15]. Furthermore, as pointed out by Shakhnovich [16], in simulations, the shape of the landscape in low-dimensional spaces is sensitive to the procedure used for dimensionality reduction, and the procedures that yield the funnel landscapes tend to be physically unrealistic.

Despite the attractive simplicity of the notion that the native conformation of a protein occupies the global minimum of *G*, it remains a hypothesis. Several lines of evidence can be and often are construed as supporting this thermodynamic hypothesis, including direct measurements of the Δ*G* of folding for multiple proteins, refolding of numerous proteins after denaturation, and spontaneous folding of proteins that were produced by complete chemical synthesis. Crucially, however, all this data pertains to a small number of small, highly stable proteins that have been studied in vitro, in isolation. Even apart from problems with the quantity and quality of this data, the question remains how generalizable these results are and how relevant are they for protein folding under native conditions, that is, in the crowded cell environment (compare panels (a) and (c) in Figure 1).

In this article, we critically assess the empirical data behind the thermodynamic hypothesis of protein folding and discuss an alternative, non-equilibrium folding hypothesis.

## 2. Review of Protein Folding

### 2.1. Experimental Data on Free Energy of Protein Folding

The Δ*G* of protein folding can be determined from denaturation-renaturation experiments under the basic assumptions that proteins are completely denatured in the well-controlled experimental conditions and that such denaturation is fully reversible. A careful examination of the methodology of these experiments, however, reveals a complicated picture, with each class of methods employed for assessing the degree of denaturation rife with its own assumptions and biases (see, for example, [17] on chemical denaturation methods assayed by spectrophotometry, [18,19] for updates on urea- and guanidinium chloride-mediated denaturation methods, and [20,21] for thermal denaturation methods and microscanning calorimetry assays). A recent discussion of the biases, sensitivity issues, and other concerns in the analysis of denaturation-renaturation data can be found in [22]. All told, the results of such experiments. that have been reported for only a handful of proteins, have led to the general consensus that Δ*G* of folding is a small negative value, that is, proteins (at least, single domain ones) fold spontaneously, but are only marginally stable reviewed in [23,24,25].

There are several reasons why, in our view, the experimental evidence in support of the thermodynamic hypothesis of folding is far less compelling than it is usually perceived to be. In particular, only in very few folding experiments, the completeness of protein unfolding at the start of the experiment has been convincingly demonstrated. Although it is often claimed that proteins in such experiments were completely denatured, a closer examination shows that typically this is an assumption rather than an experimentally validated observation. In early work (1950s–1970s), the extent of denaturation was typically assessed using indirect methods, such as circular dichroism (CD), which yields a general measure of the proportion of secondary structure in a protein, or fluorescence, which assesses the exposure of individual aromatic residues to the solute, or other, similarly indirect, approaches. However, reanalysis of a subset of cases with more advanced, direct methods has shown that proteins that have been initially characterized as completely denatured often turn out to be only partially unfolded [26]. For example, an NMR analysis of staphylococcal nuclease, the second enzyme extensively studied by Anfinsen after the seminal experiments with RNase A, has demonstrated persistence of native-like structure in the protein that was denatured in 8 M urea [27]. Subsequent NMR analysis of multiple, diverse proteins has similarly revealed preservation of extensive structure in 10 M urea [28]. Strikingly, for the paradigmatic case for the thermodynamic hypothesis, RNase A itself, advanced methods, such as small-angle X-ray scattering (SAXS) and time-resolved fluorescence energy transfer, have demonstrated that compact regions survive many thermal and chemical denaturation regimes [29]. Furthermore, it has been shown that both the degree and the character of unfolding of the same protein can substantially differ depending on the denaturation protocol (e.g., [30,31]). A computational study of protein conformers, in which backbone torsion angles were randomly varied for only 8% of the residues, while the remaining 92% of the residues remained fixed in their native conformations, has shown that the vast majority of these ensembles had end-to-end distances and mean radii of gyration that were within the range of the random-coil expectations. Therefore, it has been concluded that observation of random-coil statistics for denatured proteins cannot be taken as evidence of the absence of residual structure [32].

Measurements of Δ*G* of folding have been collected in protein thermodynamics databases, of which the most comprehensive one is ProTherm/ProThermDB. The latest release of this database [33] contains more than 30,000 entries. It would be important to determine how many records in the database are informative for estimating ranges of folding Δ*G*. We analyzed the slightly smaller, 2017 release of ProTherm (available at [34]) that contained 26,045 records representing ~700 unique wild-type proteins, of which less than 500 were annotated as reversibly denatured. Strikingly, only for 18 of these proteins, denaturation was monitored using a rigorous method, such as NMR, and for three of those the actual values of Δ*G* for the entire molecule were not reported (Supplementary Files S1 and S2). Thus, in actuality, the “vast body of curated literature” does not amount to much. Nearly all experimental data that are cited in support of the hypothesis of spontaneous refolding was obtained on a limited set of compact, stable, globular proteins. Most of these are small, single-domain monomers that are marginally stable (Δ*G* of folding between −3.5 and 7 kcal/mol) and have been shown to fold rapidly- typically, on the millisecond time scale [35]. Furthermore, the most thoroughly studied set of spontaneous refolders is enriched in extracellular proteins, often containing disulfide bonds, which are likely to dominate the fold stabilization mechanisms (see more on this below where we discuss total protein synthesis).

With all these caveats in mind, the reported Δ*G* values are within the range of −1 to −20 kcal/mol, with a Poisson-like distribution peaking around −5 kcal/mol [36]. The common range that is pervasively quoted in the literature is −5–15 kcal/mol, which is typically interpreted qualitatively as “proteins are marginally stable”, or in other words, the folding funnel is thought to be extremely shallow (e.g., [37,38,39,40]).

New opportunities to study the thermodynamics of protein folding/unfolding could be provided by single-molecule methods; for an overview of these methods as applied to protein folding, see [41,42], and for Δ*G* measurement using these methods, see [43,44,45]. However, these studies face the same major problem as bulk denaturation experiments discussed above: most proteins do not unfold completely in single-molecule manipulations, such as atomic force microscopy or optical tweezers. Almost always, there is an uncertainty about the amount of residual structure, as indicated by the fact that the stretched form of a protein is often measured to be shorter, or occasionally, paradoxically longer than the theoretically predicted length (e.g., [46,47,48]). There also indications that the theoretical length of a polypeptide chain can be sequence- and structure-dependent [48]. Taken together, these data suggest that single-molecule methods are not yet sufficiently reliable for a confident determination of the state of protein unfolding.

Overall, the survey of the experimental study of protein folding/unfolding shows that Δ*G* has been measured only for a highly biased set of a few small, compact, single domain proteins, and even for most of these, the obtained values cannot be considered reliable due to the lack of evidence of complete unfolding or, worse, presence of evidence of persisting, residual secondary structure. Even for those few proteins, for which reliable experimental data have been obtained, the negative Δ*G* values were low, in many cases, not far above the level of thermal fluctuations.

### 2.2. Chemically Synthesized Proteins Folding into Native Conformations

Another major argument in support of the thermodynamic hypothesis of folding is thought to come from experiments on proteins obtained by complete chemical synthesis. By and large, Δ*G* of folding for these proteins has not been measured directly, but is strongly believed to be negative because these proteins were produced by ligation of individual amino acids or peptides in a chemical reaction, in the complete absence of ribosomes, chaperones or other cell components, and then folded into native conformations in solution. Such spontaneous folding from the denatured form is commonly seen as direct, highly convincing evidence in support of the thermodynamic hypothesis (notably, the Nobel Prize has been awarded to Anfinsen only after the appearance of papers from Hirschmann and Merrifield groups on the complete synthesis of RnaseA [49,50]).

To assess the evidence obtained from this type of experiments, we performed a literature search on the proteins that, in the last 50 years or so, were produced by complete chemical synthesis and refolded to the biologically active form. In the set of about 60 unique proteins (not counting mutants and variants) studied in these experiments, only two were longer than 200 aa; the mean protein length in this group was 94 amino acids, which is at least 3–5-fold less than the proteome-wide mean lengths in Archaea, Bacteria, and Eukarya (Table 1). The proportions of secreted proteins and proteins containing disulfide bonds (DSB) in this dataset was several-fold higher than in the complete proteomes of various organisms (cysteine preference in these sequences is built in because modern methods of complete chemical synthesis assemble proteins from peptides, which usually requires internal cysteine residues for conjugation chemistry). Thus, recapitulating the properties of spontaneously refolded proteins discussed in the preceding section, the set of chemically synthesized proteins is heavily biased and not at all representative of real proteomes. Furthermore, there is no reliable data on those targets that were synthesized but could not be refolded. Even apart from these limitations, successful folding of chemically synthesized proteins requires non-physiological renaturation times in the hours’ range. The yields of the native conformations are often omitted from the reports, but vary widely when reported (5–48%; Supplementary File S3); the folding protocols are complex and are developed on a case-by-case basis. Thus, even for this collection of privileged proteins, folding to the active forms in vitro is not straightforward and likely proceeds differently than in vivo.

Then, there is an even deeper problem with the folding of chemically synthesized proteins as the ultimate argument for the thermodynamics hypothesis. Through the course of the chemical synthesis, these proteins remain attached to solid phase, with limited degrees of freedom for the main chain rearrangement. The structure of the Gibbs energy landscapes (or other landscapes) for such proteins and their precursor peptides, before or after they are released from the solid phase into the solution, is completely unknown. These landscapes would be important to study, not only to resolve this open question as such, but also because this might yield clues both to the mechanisms of protein folding inside cells and to the folding of primordial peptides during the early evolution of life (see discussion towards the end of this paper).

### 2.3. Refolding of Insoluble Overexpressed Proteins from Denatured Bacterial Inclusion Bodies into Soluble Active Proteins in Native Conformations

A widely used approach to protein production is based on the fact that, when a recombinant protein is overexpressed in bacterial cells, it often forms insoluble inclusion bodies that are easy to isolate from other cellular components. Such aggregates of overexpressed proteins can be collected, further purified, denatured in vitro and often can be successfully refolded into soluble, active proteins. Because of the numerous industrial applications, protein purification and refolding from bacterial inclusion bodies have been extensively studied (for overview, see [56,57,58,59]).

If indeed the proteins that are purified from inclusion bodies were shown to unfold completely and then routinely refold to the native, active conformation, this would comprise strong evidence that spontaneous protein folding is common, in accord with the thermodynamic hypothesis. However, experiments with overexpressed proteins that form inclusion bodies resulted in a key observation that suggests quite different conclusions. Typically, proteins within the aggregates that form the inclusion bodies are neither disordered nor unfolded, but have specific secondary and tertiary structures, which are substantially ordered and are often enriched in in-register beta-sheets [60,61,62,63]. Detailed analysis of the refolding process shows that some of the ordered structure is preserved throughout the purification stages ([64,65,66], reviewed in [67]). Moreover, harsh denaturing conditions tend to be detrimental for protein refolding to the native conformation, so that new protocols for unfolding–refolding under mild conditions are constantly proposed in attempts to improve the yield of functional proteins (e.g., [68,69,70]).

Thus, experiments on refolding of overexpressed proteins demonstrate the key role of the residual secondary and tertiary structures, which are generated in the first place by the cell during protein expression in vivo and apparently have to be retained by the protein for efficient refolding. Furthermore, even when refolding occurs, it barely resembles the folding processes that occur in living cells. Indeed, purification and refolding of nearly every protein requires extensive protocol development, which often includes solutions and treatments that are far from physiological conditions and refolding times that are typically much longer than the biologically relevant time scale. All these efforts notwithstanding, the yields of the refolded native proteins vary widely [57,59]. Therefore, in general, refolding of proteins from inclusion bodies cannot be counted as a showcase for spontaneous refolding of completely denatured proteins and hence does not provide clear support for the thermodynamic hypothesis.

### 2.4. Scarcity of Data on ΔG of Protein Folding Reflects Pervasive Non-Refoldability and Instability of Proteomes

A general conclusion from all of the above is that the evidence for the negative Δ*G* of folding is quite thin, at best, and that the data on the ability of proteins to refold from a completely denatured state is fragmentary and comes from small, heavily biased datasets. What causes this scarcity of data, especially for larger proteins? The principal cause appears to be quite simple: most proteins actually cannot refold once completely unfolded, but the negative results of this type, that is, failed attempts to refold proteins, are almost never published. To our knowledge, no representative samples of proteins have been studied under this angle until very recently. However, in a recent proteome-wide study, protease-resistance assay was used in combination with quantitative mass-spectrometry to show that about 50% of the proteins in *E. coli* cell lysates could not refold into their native states following chemical denaturation, even when the conditions were optimized for refolding [71]. These findings indicate that non-refoldability in vitro is a general characteristic of at least this bacterial proteome, especially, taking into consideration that the completeness of unfolding was not monitored in these experiments.

Another widely observed but often misinterpreted phenomenon is the general proteome instability, that is, pervasive spontaneous loss of native structure and activity in proteins that have been originally properly folded in the living cells. This loss of native conformation and functional activity is commonly observed both in vitro, in preparations of isolated proteins, and in vivo. Indeed, it is well known that all cells maintain elaborate proteostasis machineries that functions to repair or destroy any proteins that have irreversibly lost their active conformations [72,73].

Spontaneous protein unfolding (denaturation) in vitro is an extremely common observation which suggests that at equilibrium many if not most proteins are in unfolded conformations. Unfortunately, to our knowledge, there is no published comprehensive statistics on protein (in)stability in vitro. Studies on protein stability and approaches to stabilization are a major expense in the pharma/biotech/industrial enzymology industry, and apparently, much of the results comprise intellectual property of these companies. The physical and chemical processes that are associated with instability have been thoroughly studied for a relatively small number of proteins [74,75,76]. The principal take-home message from these experiments is that even correctly folded proteins are often not intrinsically stable, as it would have been expected if they were in a deep free energy minimum, either global or local; instead, many lose native conformation easily.

Protein engineering experiments indicate that many proteins are easily destabilized with small sequence changes. However, despite years of research, predicting the effect of mutations on protein stability remains challenging. Nevertheless, the general conclusion is that most proteins are only barely stable, such that there are many destabilizing mutations (reviewed in [77,78]). Poorly understood tradeoffs seem to exist between protein stability and solubility such that a mutation on the exterior of a protein that increases its solubility is often destabilizing [79,80,81,82]. Similarly, there are tradeoffs between protein activity and stability such that mutations that enhance enzyme activity often destabilize the protein, and vice versa, stabilizing mutations often decrease activity [83].

Over the decades, many ad hoc explanations have been given for the spontaneous unfolding, denaturation, and destabilization that proteins typically undergo. Mostly, some irreversible events are postulated to occur during unfolding, such as protein oxidation, other chemical modifications, and/or aggregation, and such secondary effects are claimed to shift the equilibrium towards the unfolded state, preventing thermodynamically driven folding [74,76,84,85]. However, few targeted studies of protein denaturation mechanisms have been published. Usually, the loss of the native conformation and consequently activity by an isolated protein is perceived as a (often major) nuisance and is rarely seen as an opportunity to study the mechanisms of irreversible denaturation, and apparently, for this reason, not much systematic research has been done in this field. A notable exception are experiments of Klibanov and colleagues on the mechanisms of amylase denaturation. In these studies, the processes involved in thermal inactivation of this enzyme were dissected, showing that denaturation (unfolding), chemical modification, and aggregation are all distinct processes separated in time, and irreversible denaturation of this enzyme precedes chemical modifications and aggregation [86,87]. Several studies on other enzymes have also demonstrated that denaturation by irreversible chain unfolding is a process distinct from protein aggregation [88,89,90].

There is a call in the literature to apply modern approaches, such as new methods of spectroscopy and mass-spectrometry, for the proteome scale analysis of protein stability [91,92,93,94], but the actual experiments of this type remain to be performed.

A rough estimate of the failure rate of attempts on isolation of proteins in the native conformations can be extracted from large-scale structural genomics projects, which publish some statistics of protein production and purification. For example, Page et al. [95] reported that of more than 1800 proteins encoded in the genome of the hyperthermophilic bacterium *Thermotoga maritima* and cloned into expression plasmids, only 539 (~29%) could be purified in the form suitable for crystallization. In the Northeast Structural Genomics Consortium project [96], 6493 proteins could be purified out of the total 16,992 expressed (34%). The New York Structural Genomics consortium has not reported consolidated statistics, but ~30% purified-to-expressed ratio seems to be a general trend across many participating projects [97]. It should be noted that in all these efforts, except for the Thermotoga case, the set of targets is strongly biased by pre-selection for predicted solubility, globularity, and evolutionary conservation. Even in these privileged sets of proteins, two-thirds could not be purified in the native conformation.

Generally, for the vast protein space, the thermodynamic parameters of protein folding and unfolding remain effectively unknown. There is no strong evidence that negative Δ*G* of folding is a general property of many proteins. On the contrary, a wealth of data seem to present evidence against this possibility, showing instead that unfolding of most proteins occurs spontaneously, whereas folding does not. Thus, protein folding appears to be a non-equilibrium process that is accompanied by free energy increase.

### 2.5. Special Features of Protein Folding In Vivo

Complex proteostasis systems operate in every cell, and malfunction of these systems leads to (often lethal) accumulation of unfolded and misfolded proteins in the cell [72,73]. Proteomics shows that more than two thirds of all proteins in yeast specifically interact with one, or often more than one, of the proteostasis systems, and more specifically, with molecular chaperones [98]. Hundreds of proteins in *E. coli* interact with the chaperone GroEL-GroES alone [99]. The most abundant proteins in eukaryotes (actin, tubulin) do not fold in vitro at all, and to fold in vivo, they require, in addition to general chaperone systems, also the specialized co-chaperone prefoldin [100,101]. Apparently, proteostasis mechanisms consume a substantial fraction of the cellular energy supply; although the estimates do not seem to achieve high precision, the fraction of the energy budget dedicated to these processes is thought to be greater than 10% [102].

Chaperone clients are classified based on how closely they interact with the chaperones (for example, obligately-dependent vs. partially-dependent clients, based on the occupancy of the client-chaperone complexes [99]) and what, specifically, do they need chaperones for some proteins aggregate in the absence of chaperones, others stay soluble but are inactive, yet others need chaperones only under stress [103,104].

How do chaperones facilitate protein folding? The dominant view is that they help client proteins to quickly reach the minimum of free energy, that is, the chaperones create conditions for the thermodynamically driven folding of a substrate protein molecule into the native conformation. Some specific mechanisms include: (1) holding the client in isolation so that it does not aggregate with other proteins and folds correctly by itself, a mechanism known as “Anfinsen’s cage” in the case of GroES/GroEL [105,106,107], (2) preventing client proteins from getting stuck in kinetic traps during folding, conceivably, via partial unfolding [108,109,110], (3) unfolding misfolded or aggregated substrates before proceeding with mechanisms (1) or (2) [111,112,113,114], (4) reshaping the folding landscape in ways different from mechanism 2, known as “kinetic assistance”, but typically not specified further [115,116,117,118].

Some chaperones, known as foldases, are ATPases, whereas others, dubbed holdases, are not [119], but the distinction does not appear to map well onto the mechanisms listed above. Indeed, some chaperones from each functional class seem to exercise mechanisms 1–4 (see, e.g., [120]), whereas some appear to combine properties of foldases and holdases, as argued for the ATP-independent chaperone trigger factor [121] as well as the ATP-dependent HSP70 [122,123].

Crucially, all proposed chaperone mechanisms are predicated on the thermodynamic hypothesis, and to our knowledge, the relevance of these mechanisms has not been tested against the alternatives. A different view of the chaperone mechanisms will be discussed below.

### 2.6. Is Protein Folding In Vivo an Active, Energy-Dependent Process?

In our view, the above discussion shows that there is very little experimental evidence that Δ*G* of folding is negative for most proteins. Conversely, a massive amount of experimental observations indicates that native conformations of proteins are only metastable. Taken together, these lines of evidence compel us, in the least, to seriously consider the possibility that, for the majority of proteins, Δ*G* of folding is positive (Figure 1d). The key implication of this hypothesis is that protein folding in vivo does not occur spontaneously, but rather, is an active, energy-dependent process.

This conceptual shift in our understanding of protein folding further implies that the ribosome itself is likely to act as a giant chaperone and the most important part of the protein folding machinery [124,125,126]. Clearly, this possibility is fully compatible with the numerous observations indicating that folding of most if not all proteins occurs co-translationally [127,128,129,130,131,132,133,134,135,136,137,138,139,140,141,142,143,144,145,146,147,148,149,150,151].

The most obvious way the ribosome could cause the increase in the Gibbs free energy that seems to accompany protein folding is by lowering the entropy of the protein by reducing the number of possible conformations of the peptide backbone. Some strained conformations with elevated enthalpy appear possible, too.

A common assumption in modeling of protein folding and in theoretical discussions is that the protein backbone can be well approximated by a freely jointed chain (FJC), so that all energy that could be applied to it would rapidly dissipate because of unrestricted rotation around each psi and phi bond. However, theoretical argument against this assumption, based on the available data on excluded volume effects and steric hindrances, has been brought up (e.g., [152]). Recently, our all-atom molecular dynamics simulations have revealed the situations when the backbone indeed is not FJC. When rotational force is applied to the protein backbone during the simulation, diverse helical peptides, despite their purported freedom to rotate about the psi and phi bonds, rapidly fold into the native structure, which remains stable [153].

It is important to recall that translation is coupled to the hydrolysis of massive amounts of GTP, but there is no clarity as to what this energy is actually expended on [154]. If Δ*G* of protein folding is positive, it appears likely that at least some fraction of the energy of GTP hydrolysis contributes to active, non-equilibrium, co-translational folding. Apart from the ribosome, other molecular players are likely to be involved in active co-translational (and “co-translocational”) folding as well, in particular, the signal recognition particle (SRP) that contains two GTPases of its own, while the role of GTP hydrolysis is no better understood than it is in the case of the ribosome [155].

The energy-coupling machine framework has been suggested also for chaperone mechanisms as an alternative to the Anfinsen’s cage. Once again, it is unclear what the energy of ATP hydrolysis by ATP-dependent chaperones is actually spent on. Most studies link the ATPase activity with rearrangements of the chaperone itself [156,157]. However, the energy balance of these reactions remains unknown, and the possibility of coupling between ATP hydrolysis and the client protein rearrangements is typically not even considered because folding is assumed to be spontaneous. In contrast, a series of studies pioneered by Lorimer, De Los Rios and Goloubinoff argue that ATP-dependent chaperones, such as HSP60, HSP70 or HSP90, might expend at least part of the energy of ATP hydrolysis to manipulate the substrate directly (“non-equilibrium activation”) although the mechanistic details remain unclear [158,159,160,161,162,163].

Apart from the empirical evidence and thermodynamic considerations, the notion of active, non-equilibrium protein folding also appears to be better compatible with the evolutionary history of the relevant cellular components than the thermodynamic hypothesis of spontaneous folding. Indeed, the ribosome, translation factors with GTPase activity, and the SRP are universal to all cellular life, and several key chaperones also are among the most highly conserved proteins. All these molecular machines are likely to antedate the Last Universal Cellular Ancestor [164,165,166]. During all the 4 billion years or so of their existence, natural selection (including purifying selection for most of this time) would have acted primarily on the foldability of proteins on these machines, rather than their ability to fold/re-fold spontaneously. Perhaps, spontaneous folding could be a factor only occasionally, in particular, for secreted proteins that have little access to chaperones once outside the cell.

### 2.7. Towards a Realistic Physical Model of Active Protein Folding

If the thermodynamic concept of protein folding generally fails, a new physical model of protein folding as an active, energy-dependent process is needed. Where to start? To begin with, a better definition of a “perfectly unfolded” protein conformation is essential. In such a fully unfolded conformation, there are no stable contacts between any two amino acids that are not adjacent in the polypeptide chain. It is currently unclear whether a perfectly unfolded conformation actually exists in vitro or in vivo for any particular protein, but this definition will be an appropriate starting point for building a physical model and recreating the folding process in silico.

Depending on the length of the polypeptide chain, there are theoretically on the order of 10^100^ perfectly unfolded conformations for each protein [167,168]; more recently, suggestions have been made for a more conservative upper bound which, however, remains astronomically high [169]. From this vast set of perfectly unfolded conformations, one can build up and arrive at all kinds of structures: active intrinsically disordered (“natively unfolded”) conformations, stable misfolded conformations, conformations that only emerge through interaction with other proteins, classic globular native conformations with hydrophobic cores, and more. What do we know about “perfectly unfolded” conformations? If we measure (calculate) Gibbs free energy for these conformations, we should observe approximately the same value for each of them because these are random conformations with no interactions other than with the solvent. Even a single contact that forms within the polypeptide chain, whether it is a short or long distance one, makes the polypeptide more compact and increases the Gibbs free energy both due to the entropy reduction resulting from limiting the degrees of freedom and to changing enthalpy if, for example, the contact is hydrophobic or ionic.

Considering that most of protein folding in vivo takes place co-translationally, while the polypeptide chain is built up one amino acid at a time, simultaneously exploring the shifting folding landscape while interacting with multiple other molecules in a crowded environment, the task of incorporating all known cellular biochemistry and structural biology into the physical model of non-equilibrium protein folding as it occurs in vivo seems daunting. Nevertheless, this goal no longer appears to be out of reach. Advanced methods for quantitative measurement of various energy inputs, molecular motions, heat transfers and other relevant quantities should provide the values, or at least the bounds, of many parameters that determine protein folding as in vivo. Such work has already started although it is notable that many crucial parameters of the relevant processes, even some basic ones, such as the translation rate, rely on estimates obtained decades ago [170,171] and refined only very recently [172].

A complementary class of approaches involves building, both in silico and in vitro, simplified artificial protein folding machines that apply various forces to the folding polypeptide in an attempt to directly manipulate the peptide backbone into the desired conformations, imposing various kinds of physical constraints on the folding process, and thus, causing shifts and introducing kinetic barriers into the folding landscape. Work in this direction has already started as well. In the next section, we provide a brief overview of several advanced techniques and some recent observations, which suggest a more sophisticated understanding of the mechanisms of protein folding than what was provided by the canonical models of spontaneous protein folding in vitro.

### 2.8. Non-Equilibrium Protein Folding: New Approaches and Recent Results

In recent years, a variety of novel experimental techniques have been applied to study co-translational and chaperone-assisted protein folding. Particularly informative are methods that can manipulate a defined single molecule using a specific force probe, such as atomic force microscope, optical tweezers, or magnetic tweezers; these methods are sometimes collectively referred to as single-molecule force spectroscopy methods (SMFS; reviewed in [173]). The SMFS methods have been recently applied to the study of co-translational folding of nascent protein chains, using “life-like” in vitro translation systems (reviewed in [149]). Although SMFS approaches have not yet reached the precision required to infer the thermodynamic parameters of protein folding (see Section 2.1 above), these approaches are well-suited to address questions about the effects of specific treatments and interactions on the folding process. Recent observations made using such methods include, for example, detection of co-translational folding intermediates, suggesting a defined folding pathway for a small domain that had been thought to fold in a two-step fashion in vitro [174], and observation of a direct accelerating and stabilizing effect of the ribosomal tunnel on the co-translational folding of another small domain [151]. Although often discussed within the conventional framework of thermodynamically-driven folding, these and similar results can be productively exploited to develop the non-equilibrium protein folding model. Furthermore, with regard to chaperone-assisted protein refolding, SMFS methods have revealed that the chaperones of the Hsp90 family use the energy of ATP hydrolysis to perform mechanical work, which is applied to compact unfolded chains against the counteracting denaturing forces [175], in an apparent contradiction to the traditionally envisaged, Anfinsen chamber-like mechanisms of chaperone action.

Another group of powerful methods are modern structural biology approaches, including cryo-electron microscopy, solid state nuclear magnetic resonance, SAXS and others, which reveal the structure of nascent polypeptide chains during protein synthesis. Many of these methods are focused on the kinetics and regulation of protein synthesis [176,177] and on the functions of nascent chain, such as sensing the state of regulatory metabolites in the environment and communicating the results to the peptidyltransferase center of the translating ribosome [178,179]. These studies also highly informative for the study of co-translational protein folding, and have already illuminated defined secondary and tertiary structures adopted by nascent peptides in the ribosome tunnel and exit vestibule [180,181,182]. In the forthcoming years, we expect to see more explicit investigation of the interactions of the nascent peptide with the peptidyl transferase center, ribosome exit tunnel, and other components of the protein folding machinery.

Computational modeling of protein folding also is taking a new direction towards a closer mimicking of the folding environment encountered by proteins in vivo. We recently reported the results of all-atom molecular dynamics simulations, in which the standard force field was augmented by the application of a mechanical force that rotated a single N-terminal amino acid of peptides, while simultaneously restricting the movements of a distal amino acid. Such directional rotation changed the peptide backbone behavior, facilitating rapid formation of native structures in several diverse alpha-helical peptides [153]. Apparently, steric clashes arising due to the forced directional rotation resulted in the behavior of the peptide backbone that no longer resembled an FJC. Further studies are needed to determine whether such an effect can be observed in single-molecule experiments in vitro as well. Other attempts to build simplified folding machines to model aspects of co-translational peptide folding in vivo include the molecular-dynamics studies of folding in a tubular chamber representing the ribosome exit tunnel, either with uncharged elastic walls or with charged walls [183,184,185,186]. Finally, sophisticated methods of visualization and analysis of the massive dynamic data on protein folding, unfolding, and refolding are also undergoing active development (see [187] for a recent review). Such methods should greatly aid our understanding of the complex mechanisms of protein folding in vivo.

## 3. Conclusions

The cornerstone assumption in the field of protein folding is that proteins spontaneously fold into their native conformations driven by negative Δ*G*. Furthermore, it is generally assumed that the native conformation of a protein is the global minimum of Gibbs free energy. However, a survey of the available data on spontaneous protein folding and refolding, in particular, for chemically synthesized and over-expressed proteins, presents little evidence in support of this thermodynamic hypothesis of folding. On the contrary, the majority of proteins appear not to be spontaneously foldable and are only marginally stable, at best. The totality of these observations along with thermodynamic considerations suggest that across the protein world, there is a wide variety of rugged, dynamic landscapes of folding free energy, resulting in a broad range of thermodynamic and kinetic stability, and refoldability of proteins. For different proteins, Δ*G* of folding can be either negative or positive, conceivably, for the majority of the proteins. Even regardless of the specific value of Δ*G*, folding of most proteins is likely to be an active, non-equilibrium, energy-dependent process. This conceptual shift in our understanding of protein folding appears to be best compatible with the extensive molecular data on the universal translation and proteostasis machineries that operate in all cells, and with the evolutionary history of these molecular machines that is traced to the earliest stages of life evolution. We believe that this change in perspective on protein folding can and should stimulate a dedicated program of theoretical, modeling, and experimental studies.

## Figures and Tables

**Table 1:** Properties of 59 proteins produced by total chemical synthesis and refolded to their active forms, as compared to the properties of whole proteomes.

	Total Chemical Synthesis ^1^	Archaea	Bacteria	Eukarya	Data Sources for Archaea, Bacteria and Eukarya
mean protein length, amino acids	94	283	320	472	[51]
% secreted	62	6–19	18–30	13 (humans)	[52,53,54]
% with DSB in the known 3-D structures	57	15	11	30	[55]

^1^ For the full data compilation from the literature, see Supplementary File S3.

## Data Availability

All data supporting reported results have been submitted as Supplementary Files to this manuscript, except the ProTherm data that have been converted by Protabit LLC with permission from ProTherm and deposited at https://github.com/protabit/protherm-conversion (accessed on 6 December 2021).

## Supplementary Materials

The following are available online at https://www.mdpi.com/article/10.3390/ijms23010521/s1.
